# ICF components of corresponding outcome measures in flexor tendon rehabilitation – a systematic review

**DOI:** 10.1186/1471-2474-9-139

**Published:** 2008-10-15

**Authors:** Renée Oltman, Gudrun Neises, Daniel Scheible, Gerhard Mehrtens, Christian Grüneberg

**Affiliations:** 1 Faculty of Economics and Media, Research Group Health and Economics, Hochschule Fresenius, University of Applied Sciences, Limburger Str. 2, 65510 Idstein, Germany; 2 Institution for Statutory Accident Insurance and Prevention in the Health and Welfare Services (BGW), Germany; 3 Faculty of Health, Research Group Health and Economics, Hochschule Fresenius, University of Applied Sciences, Limburger Str. 2, 65510 Idstein, Germany

## Abstract

**Background:**

The International Classification of Functioning, Disability and Health (ICF) delivers a holistic approach to health conditions. The objective of the present study is to provide an overview of flexor tendon rehabilitation outcome measures with respect to ICF components. Furthermore, it aims to investigate to which extent current assessments measure aspects of health according to these components primarily focussing on *activity *and *participation*.

**Methods:**

A systematic literature review was conducted to identify all studies meeting the inclusion criteria. Studies were only included if they assessed more than *body function and body structure *and referred to the ICF components *activity *and *participation*. The outcome measures were analysed and their linkage to the ICF components were investigated to examine to which degree aspects of health outcome as defined by the ICF were considered.

**Results:**

As anticipated, the application of outcome measures after flexor tendon repair is non conform. In many studies the emphasis still lies on physical impairment neglecting activity limitations and participation restrictions.

Aspects of health after flexor tendon repair could be assessed more adequately and cover patients' needs more sufficiently by choosing outcome measures which refer to all aspects of functioning.

**Conclusion:**

The ICF can help to identify aspects of health which are not being considered. The ICF can help promote further development of adequate outcome measures including activity limitation and participation restrictions by targeting patient centred goals and respecting patients' needs.

## Background

**Figure 1:**
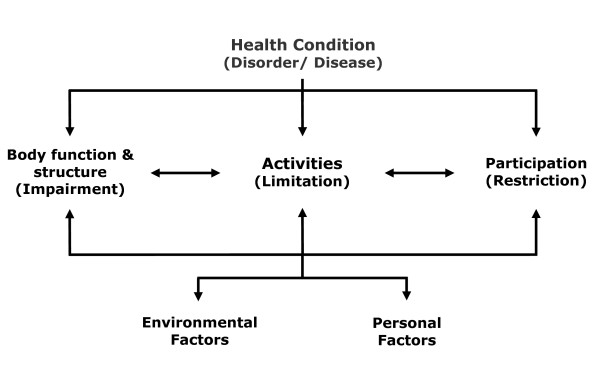
**Interaction of ICF-Concepts **[2]**.**

The International Classification of Impairments, Disabilities and Handicaps (ICIDH) introduced by the World Health Organization (WHO) in the 1980's initiated that concepts referring solely to physical impairment cannot describe the extent of health outcome after medical interventions [1]. In addition, the introduction of the International Classification of Functioning, Disability and Health (ICF) in 2001, shifted the original focus on disability and handicap to a concept including the components *body function and body structure*, *activities *and *participation*, as well as factors such as *environmental factors *and *personal factors *[1,2]. Using these components, the ICF provides a scientific framework for the classification of all health conditions (see Figure 1). Using common language it improves communication and facilitates the comparison of data concerning health outcomes. Consequences of health conditions can be classified, and moreover patient-centred rehabilitation goals can be targeted in a holistic manner [2].

Rehabilitation of flexor tendon repair remains challenging and requires experienced professionals as well as interdisciplinary approaches incorporating all health professionals concerned. Primary care of flexor tendon repair is provided by hand surgeons. The surgical treatment purpose (surgical tendon repair) aims to restore maximum flexor tendon gliding and digital function [3,4]. Post-operative management involves multi-disciplinary health care professionals and is provided by hand surgeons, as well as occupational therapists or physiotherapists specialized in hand therapy.

The treatment protocol for flexor tendon rehabilitation is dependent on the details of the injury, medical and surgical management, as well as patient-related factors and variables [3]. Three basic approaches to flexor tendon repair are described in the literature and incorporate: immobilization, early passive mobilization, and early active mobilization [3].

Even though advancements in flexor tendon repair have been made in recent years, surgeons and therapists are still confronted with functional impairment which has adverse effects on patients' activities and participation [3]. Therefore, many studies have been conducted to evaluate treatment protocols and their outcomes.

Health professionals traditionally incorporate activity limitations and participations restrictions [5]. Despite all developments regarding the change in perspective of health conditions, studies focussing on flexor tendon rehabilitation mainly describe *body function and body structure*, e.g. range of motion and the measurement of grip strength for the evaluation of flexor tendon repairs [3-22].

Furthermore, functional outcome alone does not represent the true impact of flexor tendon injuries on patients. For even minor functional loss of hand function may have adverse effects on patients and their emotions regarding their abilities to cope with tasks of daily life [23].

In this regard, outcome measures assessing *activity *and *participation *still seem under-represented. For example, Elliot & Harris reviewed outcome measures after flexor tendon repair [24]. The assessments compiled mainly included methods for measuring digital range of motion and muscle strength. In terms of the ICF, these methods exclusively refer to *body function and body structure*. The systematic review by Thien et al. investigates the evidence of rehabilitation strategies after surgery for flexor tendon injuries of the hand [25]. Only one of these studies refers to *body function and body structure *as well as to *activity *and *participation*, thus incorporating more than one ICF component [23].

The objective of the current study is to provide an overview of outcome measures for the evaluation of flexor tendon rehabilitation emphasizing the ICF components *activity *and *participation*. Therefore, our aim is to focus on previous studies which assessed more than *body function and body structure *and referred especially to the ICF components *activity *and *participation*.

## Methods

To identify the relevant literature we approached the search strategy in the following manner: Due to the nature of flexor tendon lesions it is self-evident that studies referring to flexor tendon repair and/or rehabilitation include components which can be allocated to *body function and body structure*, e.g. finger flexion, grip strength, or pain. Yet, the true health impact of flexor tendon repairs cannot be represented simply by these means. Therefore, we included studies for further analysis only if they refer to the components *activity *and *participation *(as well as to *body function and body structure*). We took into account that this strategy would considerably narrow the amount of studies to be included. By strictly focussing on these studies we anticipated to identify more relevant results, thus expecting to identify various studies covering only the ICF components body function and structure.

Our investigation search strategy was a two-step process. We searched in PubMed (Medline), The Cochrane Library, Cinahl and Embase databases, and the publisher data bases Springer and Thieme up to 11/2007, inclusive. The search strategy included the following search terms and was modified according to the specific features of the databases (see Table 1).

**Table 1:** Search terms

**search term**	**or**	**or**
flexor tendon	hand surgery	
flexor tendon	surgery	
flexor tendon	rehabilitation	
flexor tendon	Injuries/injury	interventions*

*The interventions consisted of the following search terms: physical therapy, occupational therapy, exercise, Kleinert, Duran, Washington, splint*.

To avoid extreme variations of methodological quality, only randomised controlled trials, controlled trials and clinical trials were taken into account for further examination. Publications considered were in English or German.

All studies met the following inclusion criteria:

- Deployment of outcome measures in hand rehabilitation after flexor tendon repair which can be allocated to the components *activity *and *participation *as described by the ICF

Studies containing one of the following criteria were excluded:

- Studies employing outcome measures which only refer to *body function and body structure*

- Trials with human cadavers

- Trials with animals

- Description of rehabilitation without patients and outcome measures

- Description of single cases

- Partially ruptured tendon lesions

To meet our inclusion criteria and to incorporate all studies employing outcome measures, which can be assigned to the components *activity *and *participation*, we screened all abstracts which were identified in step one. Studies which dealt with the exclusion criteria, e.g. trials with animals, were excluded from further analysis. The remaining studies were viewed in full text, whereas all utilized outcome measures were listed.

The authors allocated the listed outcome measures to the ICF components by linking these outcome measures to the most precise ICF category. The linking process was conducted according to the linking rules described by Cieza et al. [26].

The following example demonstrates the linking process: AROM (Active Range of Motion) expresses the unassisted extension and flexion of any joint. AROM is linked to chapter seven of the component *'b' *(body function). At the first level AROM is linked to *'b7 Neuromusculoskeletal and movement-related functions'*. The second level is represented by *'b710 Mobility of joint functions'*. Lower level categories do not exist and therefore *'b710' *represents the most precise category.

Accordingly, all listed outcome measures in Table 2 were linked to the appropriate category. Our results were independently confirmed with one hundred percent consensus by a colleague (AWC) who was trained in the ICF linking rules. For the purpose of our study, we chose to display our results according to the main ICF components at the body level (body function and body structures), the individual level (activities), and the social level (participation) [27]. Studies were excluded if the employed outcome measures could be allocated to the ICF component *body function and body structure*, only.

**Table 2:** Distribution of outcome measures according to the ICF components

**Study**	**Body function and structure**		**Activity**	**Participation**^2^
Adolfsson [28]	• AROM	(b710)		• Absence from work
	• Distance from the fingertip pulp to distal palmar crease	(b710)		
	• Louisville	(b710)		
	• Tsuge	(b710)		
	• Buck-Gramcko	(b710)		
	• Grip Strength (Jamar)	(b730)		

Baer [30]	• Buck-Gramcko	(b710)	• DASH^1^	
	• Quality of Composite Fist	(b710)		
	• Paresthesia in finger pulps	(b265)		
	• Grip Strength	(b730)		

Bircan [38]	• Buck-Gramcko (goniometer)	(b710)		• Return to work status
	• Grip strength (Jamar)	(b730)		
	• Key pinch (Baseline pinch gauge (FEI))	(b730)		

Friedel [23]	• Buck-Gramcko	(b710)		• Change of jobs
	• Sensitivity to cold	(b2700)		• Restrictions at work
	• Sensitivity to changes in the weather	(b279)		
	• Reduced blood circulation	(b415)		
	• Pain	(b28014)		
	• Paresthesia and numbness	(b265)		
	• Strength	(b730)		

Harris [39]	• Classification of re-repair in excellent, good, fair and poor (not further classified)	(b710)		• Occupation was recorded^4^

Ipsen [40]	• TAM	(b710)		• Period of unfitness for work (in weeks)
	• Lister et al.	(b710)		• Change of jobs (due to severe reduction of hand function)
	• Distance from the pulp to the distal palmar crease (cm)	(b710)		

Klein [32]	• Strickland/Glogovac formula	(b710)	• DASH^1^	• DASH^1^

Langlais [41]	• Tubiana's system	(b710)		• Return to same job
	• Profundus tendon function (Bichat's "claw index")	(b710)		• Time off work (in months and year)

Olivier [42]	• Buck-Gramcko	(b710)		• Work or school time loss (in weeks)

Riaz [29]	• AROM	(b710)	• Difficulties with daily activities (simple questionnaire)	• Difficulties at work (simple questionnaire)
	• Kleinert's criteria	(b710)		
	• Scar sensitivity (simple questionnaire)	(b279)		• Return to work status
	• Cold sensitivity (simple questionnaire)	(b2700)		
	• Grip strength (Jamar)	(b730)		

Su [31]	• Strickland's revised score	(b710)	• DASH^1^	
	• Pain (verbal scale 0–10)	(b28014)		
	• Swelling (in mm with circumference gauge) (in mm with circumference gauge)	(n.i.^3^)		
	• Neurologic recovery (Semmes-Weinstein monofilaments)	(b279)		
	• Grip strength (Jamar)	(b730)		
	• Pinch strength (Jamar)	(b730)		

1) The DASH Outcome Measure has recently been linked to the ICF [39] and was therefore only allocated to the components in the current table.

2) Work related information is not further classified because the methods of assessment were not further specified or not standardized, yet may be linked to d850.

3) not identified

4) Participation restrictions were not further specified in this study. Yet, due to the importance of occupational information for rehabilitation this study was included.

**Figure 2:**
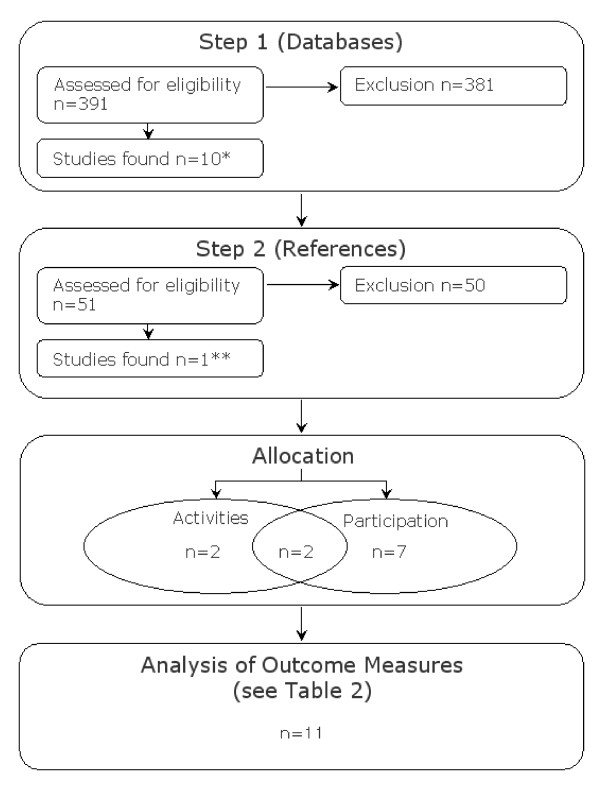
**Process of Selection**. * Studies include references [23,29-32,38,39,41,42]. ** Studies include references [28].

In step one, our inclusion and exclusion criteria allowed us to incorporate ten studies for further examination. The limited amount of studies included can be explained by our inclusion and exclusion criteria, to strictly focus on studies covering the components *activity *and *participation *(in addition to *body function and structure*) and more importantly, due to the levels of methodological quality we had chosen to adhere to. In the secondary process of selection (Figure 2, step 2) we checked the reference lists of the ten previously selected studies. This step allowed us to verify our search strategy and revealed that in step one we had failed to identify one study meeting our inclusion criteria, the study by Adolfsson et al. [28]. We included this article for further examination, because this study was originally identified through our search terms in step one (see Table 1), underlining that we had identified the relevant articles with respect to our search strategy. Altogether, as presented in Figure 2, eleven studies were enclosed for further examination.

## Results

### General results

The selected studies were published between 1988 and 2005. The analysis of the selected studies reveals that numerous factors have impact on outcome after flexor tendon repair e.g. age, gender, affected zones, concomitant injuries, timing of repair, suture technique, or post-operative rehabilitation programmes. Not only the extent to which these factors are being assessed vary remarkably, their application is non-uniform as well.

Therefore, as projected, we listed and analysed the outcome measures of flexor tendon rehabilitation and displayed their linkage to the ICF components to investigate to which degree these components are being considered, hence to what degree aspects of health outcome as defined by the ICF are being represented (see Table 2).

### Outcome measures

With respect to the ICF, the employed assessments within the identified articles are linked to the ICF components (and their categories). The linking process was conducted according to the ICF rules described by Cieza et al. [26]. The results are displayed in Table 2 which establishes a basis for comparison of outcome measures and, in addition, allows the representation of various health aspects within the assessments (see Table 2).

### Body function and structure

Even though our review focuses on *activity *and *participation *we chose to display outcome measures referring to *body function and body structure *as well. Thus demonstrating the quantitative difference between outcome measures referring to *body function and body structure *and outcome measures referring to *activity *and *participation*. All outcome measures at body level were linked to *body function *('b').

Evaluation of outcome after flexor tendon repair in terms of *body function and body structure *is most commonly assessed by classifications which categorize results from excellent, good, fair to poor, e.g. the classification system by Buck-Gramcko, Strickland/Glogovac, TAM, etc. (see Table 2 and Table 3).

**Table 3:** Short overview of specific outcome measures

**Outcome Measure**	**Short description**
AROM	Active Range of Motion measures active extension and flexion of different joints

TAM	Total Active Motion measures extension and flexion of the whole finger

Bruck-Gramcko	*

Lister et al.	*

Louisville	*

Kleinert's criteria	*

Tubiana's system	*

Tsuge	*

Strickland's revised score	*

Strickland/Glogovac formula	*

Profundus tendon function (Bichat's „claw index”)	Evaluation of profundus tendon function [41]

Grip Strength (Jamar)	Hydraulic measurement of grip strength in kg or pds

Pinch strength (Jamar)	Hydraulic measurement of pinch strength in kg or pds

Key Pinch (Baseline pinch gauge)	Hydraulic measurement of key pinch strength in kg or pds

Neurologic recovery (Semmes Weinstein monofilaments)	Sensory evaluation test

*Various grading systems and formulas have been developed to evaluate the outcome of flexor tendon repairs. The functional results usually are expressed as "excellent", "good", "fair" or "poor".

Further outcome measures include AROM, distance from the fingertip pulp to distal palmar crease, quality of composite fist, pinch and grip strength, pain, swelling, neurological recovery, and reduced blood circulation.

Additional outcome measures, which can be allocated to the ICF component *body function and structure*, include the evaluation of sensation. These outcome measures vary from simple questionnaires [29] and non-standardized evaluations [23,30] to standardized evaluation methods [31] (see Table 2).

These outcome measures address aspects of physical impairment and do not attribute to the components *activity *and *participation*. For the purpose of comparison they are listed in Table 2.

### Activities and Participation

The description of activity limitations and participation restrictions reflects the magnitude of health disturbances in various areas of life and are displayed separately in Table 2.

Outcome measures which can be linked to the component *activity *include non-standardized questionnaires assessing difficulties with daily activities [29] and *The Disability of Arm, Shoulder and Hand Outcome Measure *(DASH) [30-32].

The DASH questionnaire which was first published in 1996, is a specific outcome measure which addresses *activity *and *participation *as well as *body function *and has found employment in flexor tendon rehabilitation. It has recently been linked to the ICF framework. The authors concluded, that not all domains and categories of the ICF are covered by the DASH Outcome Measure, and may need to be supplemented by additional instruments [33].

The DASH Outcome Measure is the only standardized assessment instrument which was applied in recent studies [30-32]. However, only one of these studies further specifies if the optional modules (sports category and work category) were assessed, despite the relevance of participation restrictions in work related domains after flexor tendon repair [32].

Work related information, which may be linked to the ICF component *participation *includes the DASH Outcome Measure (work module), occupational information, return to work/school status, restrictions at work, difficulties at work, change of employment, period of unfitness for work, absence from work, and time off work (see Table 2). In regard to participation restrictions and activity limitations the obtained information from the optional DASH module is more superior than quantified time off work [34].

In addition, the identified studies demonstrate a lack of patient orientated assessments. Apart from the DASH Outcome Measure, two studies include subjective assessments. For example, Adolfsson et al. evaluated subjective hand function employing a Visual Analogue Scale (VAS) in order to compare results [28]. Friedel et al. evaluated subjective hand function using a non-standardized questionnaire (grading system not further specified) and compared these to the objective functional results (grading system by Buck-Gramcko) [23]. The authors came to the conclusion that only 40% of the subjective results correspond to the objective results. In comparison to the objective results, 26% of the population over-evaluated their results subjectively, and 21% under-evaluated their results [23].

## Discussion

The aim of this study was to provide an overview of outcome measures utilized in flexor tendon rehabilitation with respect to ICF components and to investigate the extent to which current assessments measure aspects of health according to the components *activity *and *participation*.

Even though health professionals traditionally incorporate activity limitations and participation restrictions, health outcomes typically refer to physical impairment [1,5]. This finding is confirmed by the present study on flexor tendon injuries in which all utilized outcome measures of the selected studies are listed and allocated to the components *body function and body structure*, as well as to *activity *and *participation*. The amount of outcome measures to evaluate *body function and body structure *clearly outnumber the amount of outcome measures utilized to assess *activity *and *participation*. These results point out that many studies on flexor tendon injuries still emphasize physical impairment (*body function and body structure*), neglecting activity limitations and participation restrictions.

However, possible consequences of activity limitations and participation restriction can be demonstrated by a finding in the study by Gault, in which excellent and good functional results at the level of body function and body structure do not necessarily induce complete recovery [35]. Despite excellent and good scorings (Buck-Gramcko criteria) two men in this study did not return to their former jobs as a result of loss of grip strength. In terms of the ICF, grip strength measurements (assessed in kilograms or pounds) are linked to body function (b730). Yet, as described in the above study [35], loss of grip strength may result in activity limitations, e.g. in grasping (d4401) or in manipulating (d4402), resulting in participation restrictions such as restrictions at work or even loss of work (d850). This finding emphasizes the importance of taking an holistic approach to health conditions in order to assess the full extent of health outcomes. Furthermore, as stated by Friedel et al. even a minor loss of hand function may lead to emotional consequences accentuating the need for outcome measures assessing more than physical impairment [23].

The examples above demonstrate that the ICF can assist in identifying aspects of health which are not being considered. The ICF can help promote further development of adequate outcome measures including activity limitations and participation restrictions targeting patient-centred goals and respecting patients' needs.

A means of approaching health conditions in a holistic manner is the employment of ICF core sets. ICF core sets represent all relevant ICF categories for a described health condition [36], e.g. flexor tendon repair and rehabilitation. To date, a flexor tendon core set has yet to be developed and its necessity is underlined by our findings. A limitation of this review is the restricted amount of studies which were included for further analysis. Nevertheless, by strictly including studies encompassing the ICF components *activity *and *participation *we shifted the focus from functional outcomes, to aspects of functional health, which call for more attention and can be represented by the components *activity *and *participation*.

Although we excluded studies which utilize outcome measures solely referring to *body function and structure*, these outcome measures clearly outnumber the outcome measures referring to *activity *and *participation*.

Due to our inclusion criteria only studies with a high level of evidence were included for further analysis. Therefore, relevant studies with different study designs, other than studies of medical or surgical origin, and possibly incorporating activity and participation may have been excluded. However, we had scanned additional studies which did not comply with our inclusion criteria (lower level of methodological quality) and came to the same conclusion, i.e. that participation restrictions and activity limitations are being neglected.

Therefore, future research should focus on outcome measures which additionally assess activity limitations and participation restrictions.

## Conclusion

The ICF provides a holistic approach to any health condition. With its framework we can investigate to which extent outcome measures evaluate the full scope of health aspects. Furthermore, the ICF can help to identify which health aspects are not yet being considered. Moreover, it can help promote further development of adequate outcome measures which respect patients' needs after flexor tendon repair.

Furthermore, the application of ICF-oriented instruments, e.g. the Rehabilitation Problem-Solving Form (RPS-Form) [27], which is a patient-centred evaluation tool, addresses the patients' perspective and their needs in rehabilitation.

Thus, the implementation of the ICF for evaluation of flexor tendon rehabilitation can support meeting the postulation made in former studies [1,24,37]: To reflect to which extent current assessments adequately measure aspects of health and to operationalize and assess ICF components.

Furthermore, the development of a core set for flexor tendon repair and rehabilitation will help make results comparable, thereby measuring all aspects of health after flexor tendon repair.

## Competing interests

The authors declare that they have no competing interests.

## Authors' contributions

RO and CG carried out the search, review and analysis of studies and drafted the manuscript together with DS. GN and GM helped drafting the manuscript an revised its contents critically. All authors read and approved the manuscript.

## Pre-publication history

The pre-publication history for this paper can be accessed here:


